# *Levo*-Corydalmine Alleviates Neuropathic Cancer Pain Induced by Tumor Compression via the CCL2/CCR2 Pathway

**DOI:** 10.3390/molecules22060937

**Published:** 2017-06-06

**Authors:** Yahui Hu, Nandani Darshika Kodithuwakku, Lin Zhou, Chengyuan Li, Dan Han, Weirong Fang, Jihua Liu, Yunman Li

**Affiliations:** 1 State Key Laboratory of Natural Medicines, Department of Physiology, China Pharmaceutical University, Nanjing 210009, China; huyahui324@163.com (Y.H.); 14211010262@stu.cpu.edu.cn (L.Z.); lichengyuan_cpu@163.com (C.L.); handanjelly@126.com (D.H.); weirongfang@163.com (W.F.); 2 Institute of Indigenous Medicine, University of Colombo, Rajagiriya 11600, Sri Lanka; darshi_ko@yahoo.com; 3 State Key Laboratory of Natural Medicines, School of Traditional Chinese Medicine, China Pharmaceutical University, Nanjing 210009, China

**Keywords:** microglia, astrocytes, tumor compression-induced pain (TCIP), CCL2, CCR2, inflammatory cytokines, *levo*-corydalmine (*l*-CDL)

## Abstract

*Background*: Tumor compression-induced pain (TCIP) is a complex pathological cancer pain. Spinal glial cells play a critical role in maintenance of cancer pain by releasing proinflammatory cytokines and chemokines. In this study, we verified the role of *levo*-corydalmine (*l*-CDL) on TCIP. *Methods*: Spontaneous pain, paw withdrawal threshold and latency were assessed using TCIP mouse model. Immunofluorescence was used to identify the reactions of glia. RT-PCR and western blot or ELISA were used to determine mRNA or protein expression of tumor necrosis factor-α (TNF-α), interlukin-1β (IL-1β), CC chemokine ligand 2 (CCL2) and chemotactic cytokine receptor 2 (CCR2) in vivo and in vitro. *Results*: *l*-CDL significantly attenuated TCIP hypersensitivity, accompanying with downregulation of TNF-α and IL-1β expression levels and declined astrocytes and microglial activation. It also significantly decreased the expression of the mRNA and protein level for CCL2 and CCR2. Further, *l*-CDL could suppress TNF-α-induced astrocytes activation and IL-1β expression through downregulating the CCL2/CCR2. Besides, CCL2-induced BV-microglia activation and inflammatory factors secretion were suppressed by *l*-CDL via CCR2. *Conclusions*: Suppression of CCL2/CCR2 by *l*-CDL may contribute to alleviate TCIP, offering an alternative medication for TCIP.

## 1. Introduction

Tumor compression-induced pain (TCIP) is a complex pathological process, which might seriously disrupt a patient’s quality of life. About 70–80% of cancer patients suffer from uncontrolled pain of moderate-to-severe intensity in advanced stages of the disease [1]. Cancer cells produce mediators that recruit and affect other cells within the cancer microenvironment, including nerves and immune cells [2]. Increasing preclinical studies provided ample evidence to prove that TCIP is a unique form of pathologic pain and it might share similar physiological and pathological changes with neuropathic pain and inflammatory pain [3,4,5]. Thus, TCIP is multifactorial and characterized by diverse pathophysiological mechanisms consisting of nociceptive, neuropathic, and mixed mechanisms in etiology [6]. Therefore, well-accepted animal model of cancer pain will propel preclinical studies to a new era of cancer therapeutics development [7].

Several murine models of direct inoculation of compatible murine cancer cells have been developed in recent studies [8,9], such as bone cancer pain models [10], chemotherapy-induced peripheral neuropathy (CIPN) model [11,12,13,14], and cancer invasion pain model [15]. However, only few studies were reported about the underlying mechanisms of cancer pain which is induced by tumor compression models.

Accumulating evidence suggests that activation of spinal astrocytes and microglia contributes to the development of pathological pain [16,17]. Activated glia leads to upregulation of glial markers such as ionized calcium binding adapter molecule-1 (IBA1) [18,19] and glial fibrillary acidic protein (GFAP) and/or morphological changes, including hypertrophy, proliferation, and modifications of glial networks and synthesis and release of glial mediators (e.g., cytokines, chemokines, growth factors, and proteases) to the extracellular space [20,21,22].

Many studies indicate that CC chemokine ligand 2 (CCL2 or monocyte chemoattractant protein-1), and its receptor chemotactic cytokine receptor 2 (CCR2) are involved in the maintenance of neuropathic pain [23] and inflammatory pain [24]. Recent findings demonstrate that the CCL2/CCR2 axis play a major role in the pathogenesis of cancer pain [25]. Though, it was still unclear about the effect of the CCL2/CCR2 on TCIP model.

**Figure 1:**
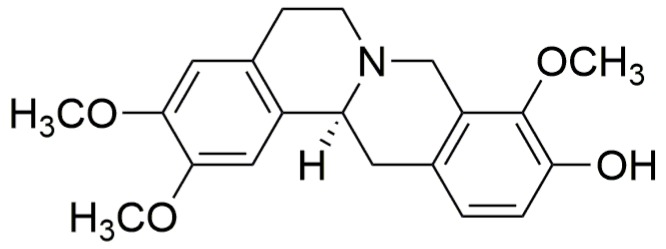
Chemical structure of *levo*-corydalmine (*l*-CDL).

Clinically, the combination oxycodone and acetaminophen (O&A) is a combined opioid/non-opioid pain reliever which relief the moderate to severe pain [26]. A recent study has shown that administration of *levo*-tetrahydropalmatine (*l*-THP) [27] at the dose of 60 mg/kg significantly prevented and/or reversed bone cancer-related pain behaviors, and inhibited tumor cell implantation-induced activation of microglial cells and the increased levels of tumor necrosis factor (TNF)-α and interleukine (IL)-18 [18]. Our previous studies showed that *levo*-corydalmine (*l*-CDL) (Figure 1), which is a THP’s desmethyl metabolite exhibited better analgesic effects and fewer side effects than *l*-THP. However, it has not yet been reported whether *l*-CDL would have the modulatory effects toward TCIP.

In the present study, a TCIP model was performed with gradual compression of a nerve by a malignant tumor, aiming to explore the role of *l*-CDL in attenuating pain hypersensitivity induced by tumor compression. Further, we investigated the changes of CCL2/CCR2 level and spinal glial cells reactivity in vivo and in vitro.

## 2. Results

### 2.1. l-CDL Attenuated Tumor Compression-Induced Pain Hypersensitivity

**Figure 2:**
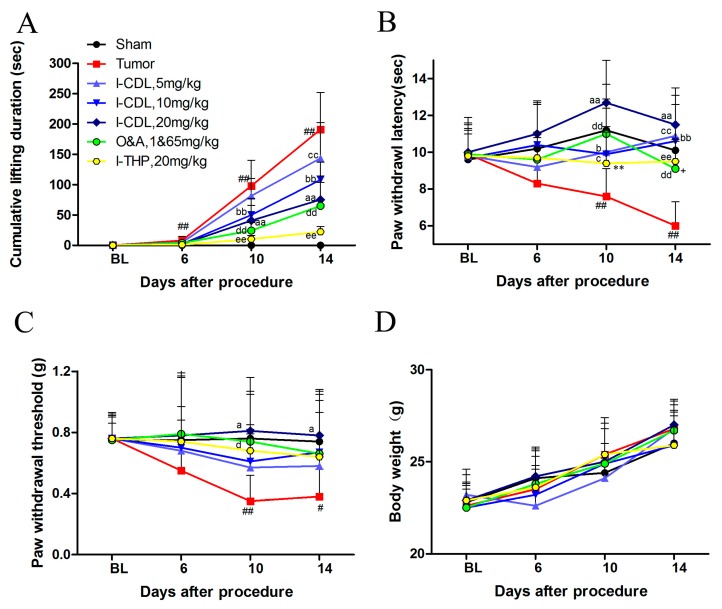
*l*-CDL attenuates tumor compression-induced cancer pain hypersensitivity. (**A**) Time course of cumulative lifting duration in mice; (**B**) *l*-CDL attenuated paw withdrawal latency of the hind paw; (**C**) *l*-CDL attenuated paw withdrawal threshold of the hind paw; (**D**) The change of body weight after the surgery. Two-way ANOVA followed by the Tukey′s post hoc test was used: ^#^ *p* < 0.05 and ^##^ *p* < 0.01, tumor vs. sham group; ^a^ *p* < 0.05 and ^aa^ *p* < 0.01, *l*-CDL, 20 mg/kg vs. tumor group; ^b^ *p* < 0.05 and ^bb^ *p* < 0.01, *l*-CDL, 10 mg/kg vs. tumor group; ^c^ *p* < 0.05 and ^cc^ *p* < 0.01, *l*-CDL, 5 mg/kg vs. tumor group; ^d^ *p* < 0.05 and ^dd^ *p* < 0.01, O&A vs. tumor group; ^ee^ *p* < 0.01, *l*-THP vs. tumor group; ** *p* < 0.01, *l*-CDL, 20 mg/kg vs. *l*-THP group; ^+^ *p* < 0.05, *l*-CDL, 20 mg/kg vs. O&A group (mean ± S.D., *n* = 7).

After inoculation, cumulative duration of lifting in the tumor group was significantly increased at day 10 (*p* < 0.01) and persisted through day 14 (*p* < 0.01) compared with sham group. Repeated intragastric use of *l*-CDL (10 mg/kg, 20 mg/kg) was significantly decreased the time course of cumulative lifting duration (*l*-CDL, 10 mg/kg versus tumor, *p* < 0.01; *l*-CDL, 20mg/kg versus tumor, *p* < 0.01; O&A versus tumor, *p* < 0.01; *l*-THP versus tumor, *p* < 0.01). However, statistical differences were significant for the cumulative duration of lifting between tumor and all treatment groups at day 14 (all doses of *l*-CDL versus tumor, *p* < 0.01; O&A versus tumor, *p* < 0.01; *l*-THP versus tumor, *p* < 0.01) (Figure 2A). A significant change in paw withdrawal latency in response to heat stimulus was observed after inoculation on the day 10 (tumor versus sham, *p* < 0.01) and further decreased by day 14 (tumor versus sham, *p* < 0.01) in the tumor group. Compared with tumor group, all the treatment groups suppressed the thermal hyperalgesia effectively at day 10 and day 14 (all doses of *l*-CDL versus tumor, *p* < 0.05, *p* < 0.01; O&A versus tumor, *p* < 0.01; *l*-THP versus tumor, *p* < 0.01). *l*-CDL at the dose of 20 mg/kg possessed stronger ability than both of O&A group and *l*-THP group in alleviating thermal hyperalgesia (day 10, *l*-CDL, 20 mg/kg versus *l*-THP, *p* < 0.01; day 14, *l*-CDL, 20 mg/kg versus O&A, *p* < 0.05) (Figure 2B). In the tumor group, paw withdrawal threshold was significantly decreased (tumor versus sham, *p* < 0.01) after inoculation at day 10 and maintained at a lower level at day 14 (tumor versus sham, *p* < 0.01). *l*-CDL (20 mg/kg) and O&A were significantly attenuated tumor induced mechanical allodynia compared with tumor group (day 10, *l*-CDL, 20 mg/kg versus tumor, *p* < 0.05; O&A versus tumor, *p* < 0.05; day 14, *l*-CDL, 20 mg/kg versus tumor, *p* < 0.05) (Figure 2C).The overall conditions of mice in all groups were good and the body weight was gradually increased after inoculation of tumor cells. No significant change in the body weight was observed among the groups during the study (Figure 2D).

### 2.2. l-CDL Decreased the Level of Tumor Compression-Induced TNF-α and IL-1β Expression in Serum and Spinal Cord

**Figure 3:**
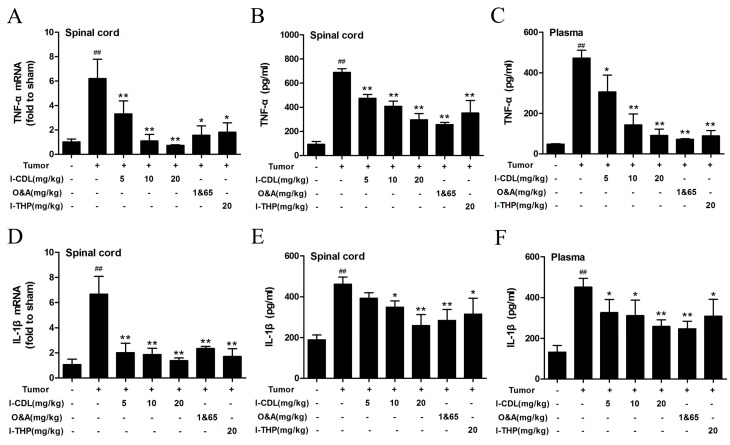
*l*-CDL attenuated tumor compression-induced TNF-α and IL-1β expression in serum and spinal cord. (**A**) The expression of TNF-α mRNA in the spinal cord; (**B**) The level of TNF-α protein in the spinal cord; (**C**) The level of TNF-α protein in serum; (**D**) The expression of IL-1β mRNA in the spinal cord; (**E**) The level of IL-1β protein in the spinal cord; (**F**) The level of IL-1β protein in serum. ^##^ *p* < 0.01 vs. sham group. * *p* < 0.05, ** *p* < 0.01 vs. tumor group. (mean ± S.D., *n* = 3).

According to the RT-PCR and ELISA results, the tumor group exhibited significantly higher levels of TNF-α and IL-1β in spinal cord and serum compared with sham group (*p* < 0.01). *l*-CDL treatment at the dose of 10 mg/kg or 20 mg/kg group significantly decreased the expression of TNF-α mRNA in spinal cord compared with the tumor group (*p* < 0.01), which showed stronger ability than O&A and *l*-THP (Figure 3A). The level of TNF-α in all doses of *l*-CDL treatment groups was significantly lower than the tumor group and dose-dependent (*p* < 0.05, *p* < 0.01) (Figure 3B,C). All the doses of *l*-CDL effectively suppressed the expressions of IL-1β mRNA in spinal cord effectively (Figure 3D). *l*-CDL 10 mg/kg or 20 mg/kg group significantly decreased the level of IL-1β in spinal cord and serum compared with the tumor group (*p* < 0.05, *p* < 0.01) (Figure 3E,F).

### 2.3. l-CDL Attenuated Tumor Compression-Induced Glial Cells Activation in the Spinal Cord

**Figure 4:**
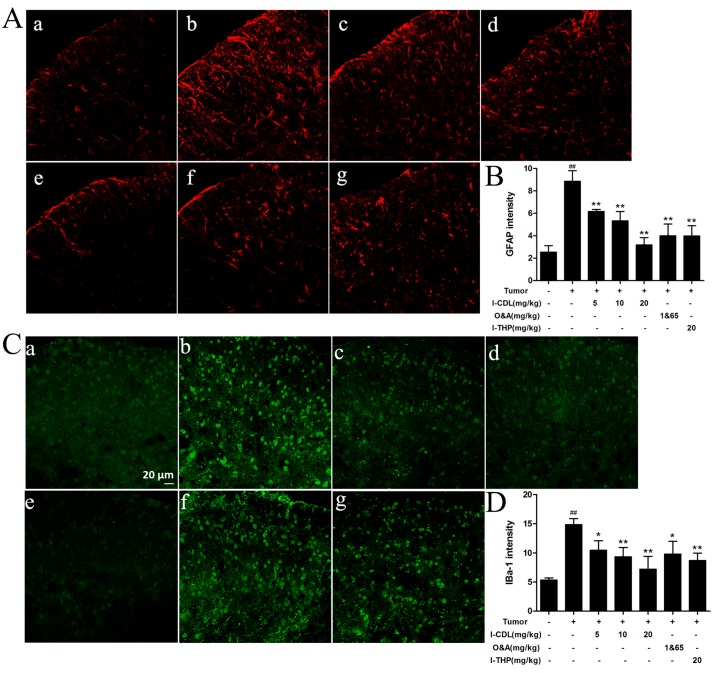
Effect of *l*-CDL on glial cells activation in the spinal cord. (**A**) The expression of GFAP in spinal dorsal horn; (**B**) Graph showing the mean fluorescence intensity for GFAP; (**C**) The expression of IBA-1 in spinal dorsal horn; (**D**) Graph showing the mean fluorescence intensity for IBA-1. (**a**) Sham group; (**b**) Tumor group; (**c**) *l*-CDL 5 mg/kg group; (**d**) *l*-CDL 10 mg/kg group; (**e**) *l*-CDL 20 mg/kg group; (**f**) O&A 1&65 m/kg group (**g**) *l*-THP 20 mg/kg group; ^##^ *p* < 0.01 vs. the sham group; * *p* < 0.05, ** *p* < 0.01 vs. the tumor group. Scale bar = 20 μm (mean ± S.D., *n* = 3).

The mean fluorescence intensity of spinal GFAP and IBA-1 immunoreactivity were greatly increased in the tumor group compared with the sham group (*p* < 0.01). All doses of *l*-CDL, O&A and *l*-THP suppressed the activation of spinal microglia and astrocytes efficiently, as demonstrated by the decreased mean fluorescence intensity of the GFAP and IBA-1 (*p* < 0.05, *p* < 0.01) (Figure 4).

### 2.4. l-CDL Attenuated Tumor Compression-Induced CCL2 and CCR2 Expression in the Spinal Segments

**Figure 5:**
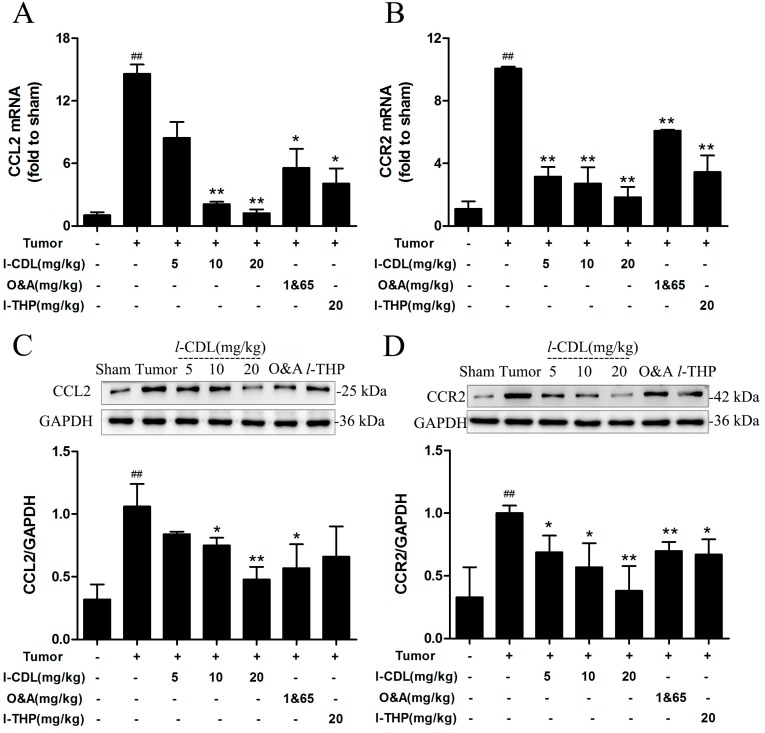
***l***-CDL attenuated tumor compression-induced CCL2 and CCR2 expression in the spinal cord. (**A**) The expression of CCL2 mRNA in the spinal cord; (**B**) The expression of CCR2 mRNA in the spinal cord; (**C**)The level of CCL2 protein in the spinal cord; (**D**) The level of CCR2 protein in the spinal cord. ^##^ *p* < 0.01 vs. the sham group; * *p* < 0.05, ** *p* < 0.01 vs. the tumor group (mean ± S.D., *n* = 3).

After tumor inoculation, the expression of mRNA and protein levels for CCL2 and CCR2 in tumor group were significantly increased compared to the sham group (*p* < 0.01). The CCL2 mRNA and protein expression were significantly reduced by *l*-CDL treatment at the dose of 10 mg/kg or 20 mg/kg group in comparison with tumor group (*p* < 0.05, *p* < 0.01) (Figure 5A,C), and possessed stronger ability than O&A group and *l*-THP group in decreasing the expression of mRNA for CCL2 levels (Figure 5A). All doses of *l*-CDL showed stronger ability than O&A and *l*-THP in decreasing the expression of mRNA for CCR2 levels (Figure 5B). All treatment groups presented a significantly lower level of CCR2 protein expression compared with tumor group (*p* < 0.05) (Figure 5D).

### 2.5. l-CDL Suppressed the TNF-α-Induced Astrocyte Activation and Downregulated CCL2/CCR2 in the Astrocyte

**Figure 6:**
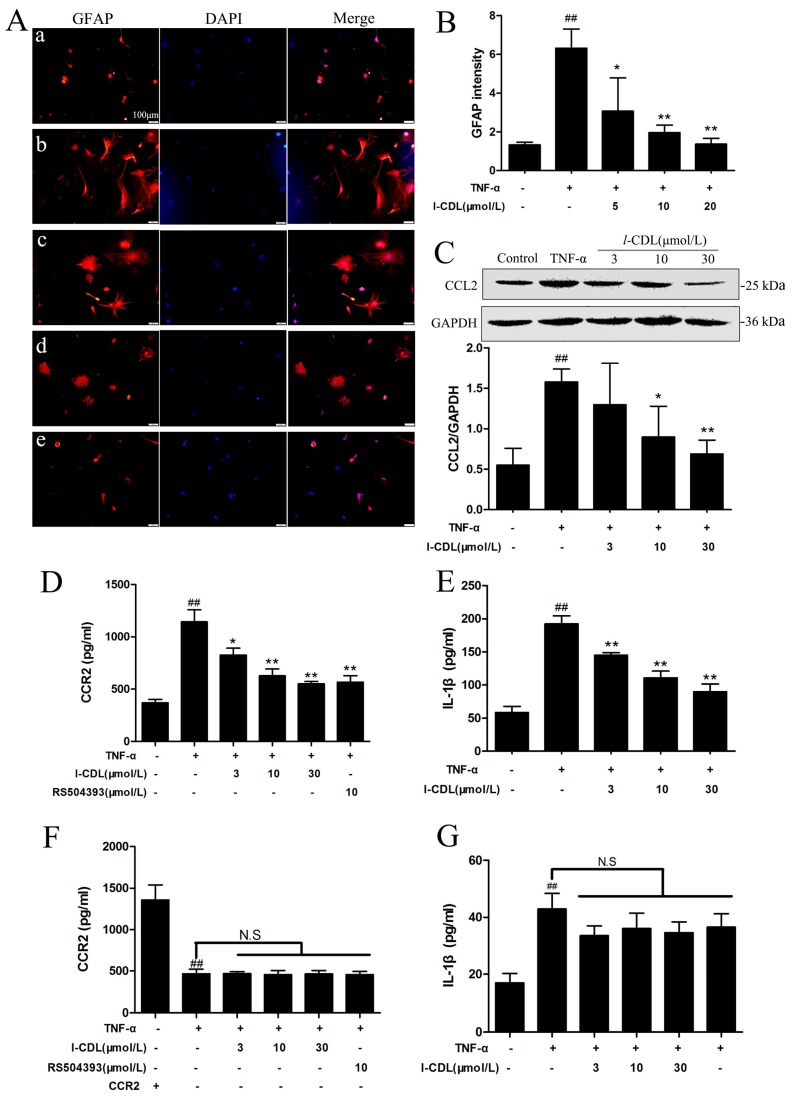
The effect of *l*-CDL on CCL2/CCR2 signal pathway in the astrocytes after pre-treatment with TNF-α. Astrocytes were pre-treated with TNF-α (10 μM) for 2 h, then incubated with *l*-CDL (3 μM, 10 μM, 30 μM) for 2 h. (**A**) Astrocytes were stained with GFAP antibody. Expression of GFAP expression (red) in activated astrocytes as visualized by fluorescence microscope. The blue staining represents DAPI. (**a**) control; (**b**) TNF-α (10 ng/mL); (**c**) TNF-α (10 ng/mL) + *l*-CDL (3 μM); (**d**) TNF-α (10 ng/mL) + *l*-CDL (10 μM); (**e**) TNF-α (10 ng/mL) + *l*-CDL (30 μM). Scale bar = 100 μm; (**B**) Graph showing the mean fluorescence intensity for GFAP; (**C**) Levels of CCL2 detected by western blotting; quantified and normalized to GAPDH levels. Each value was then expressed relative to the control, which was set 1; (**D**) The level of CCR2 protein in astrocytes (**E**) The level of IL-1β in cell culture supernatant; (**F**) Effects of CCR2 siRNA on CCR2 expression level; (**G**) Effects of *l*-CDL on IL-1β level in cell culture supernatant under the condition of CCR2 gene silencing. ^##^ *p* < 0.01 vs. the control group; * *p* < 0.05, ** *p* < 0.01 vs. the TNF-α group, N.S, no significance (mean ± S.D., *n* = 3).

Immunofluorescence result showed all concentrations of *l*-CDL treatment groups suppressed the activation of TNF-α-induced astrocytes efficiently and concentration-dependently, as demonstrated by the decreased mean fluorescence intensity of the GFAP (*p* < 0.05, *p* < 0.01) (Figure 6A,B). *l*-CDL treatment groups at the concentration of 10 μM and 30 μM were inhibited the expression of CCL2 significantly (*p* < 0.05, *p* < 0.01) (Figure 6C). All concentrations of *l*-CDL markedly reduced the level of CCR2 and IL-1β expression in dose-dependent manner (*p* < 0.05, *p* < 0.01) (Figure 6D,E). After the transfection, all *l*-CDL treatment groups incubated with CCR2 siRNA expressed lower level of CCR2 protein compared with control group, which demonstrated that CCR2 gene silencing was stable (Figure 6F). Astrocytes in TNF-α + CCR2 silencing group increased the expression of IL-1β significantly compared with sham group. Under the condition of TNF-α + CCR2 gene silencing, *l*-CDL did not regulate the expression of IL-1β (Figure 6G).

### 2.6. l-CDL Suppressed the CCL2-Induced BV-Microglia activation, Down-Regulated CCR2 Expression, Reduced TNF-α and IL-1β Secretion

**Figure 7:**
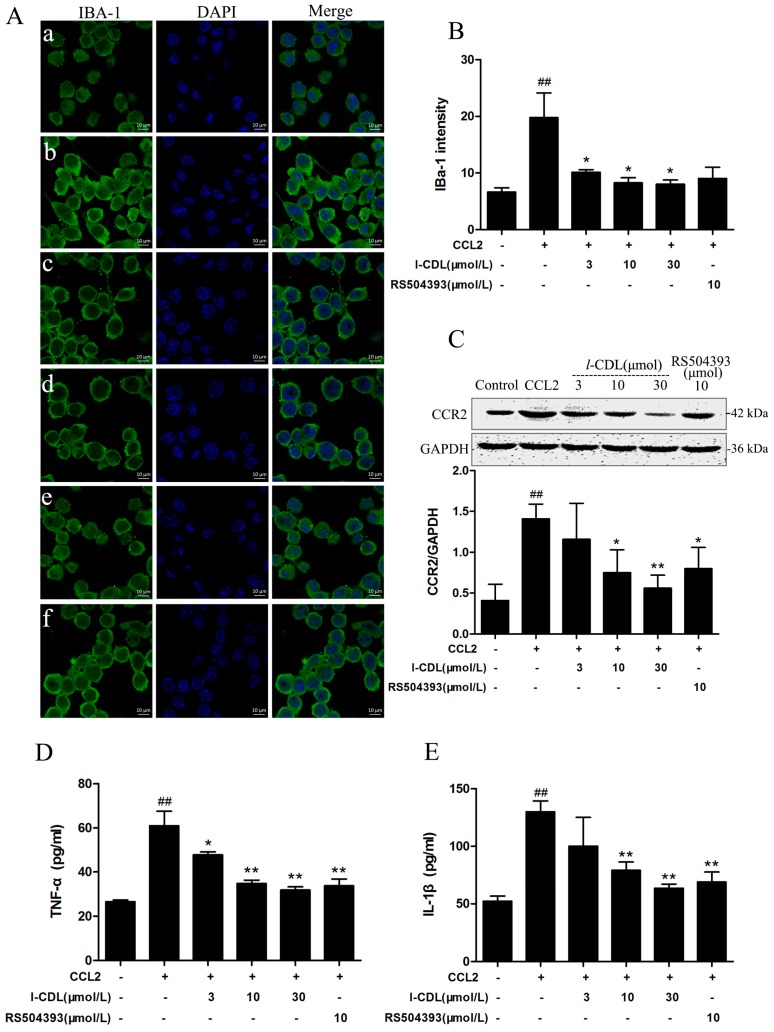
The effect of *l*-CDL on CCL2/CCR2 signal pathway in BV-microglia after pre-treatment with CCL2. BV-microglia were pre-treated with CCL2 (3 ng/mL) for 2 h, then incubated with *l*-CDL (3 μM, 10 μM, 30 μM) and RS504393 (10 μM) for 2 h. (**A**) BV-microglia stained with IBA-1 antibody. IBA-1 expression (green) in activated BV-microglia as observed using confocal microscopy. The blue staining represents DAPI. Scale bar = 10 μm. (**a**) Control; (**b**) CCL2 (3 ng/mL); (**c**) CCL2 (3 ng/mL) + *l*-CDL (3 μM); (**d**) CCL2 (3 ng/mL) + *l*-CDL (10 μM); (**e**) CCL2 (3 ng/mL) + *l*-CDL (30 μM); (**f**) CCL2 (3 ng/mL) + RS504393 (10 μM); (**B**) Graph showing the mean fluorescence intensity for IBA-1; (**C**) Levels of CCR2 detected by western blotting, quantified and normalized to GAPDH levels. Each value was then expressed relative to the control, which was set to 1; (**D**) The level of TNF-α in cell culture supernatant; (**E**) The level of IL-1β in cell culture supernatant. ^##^ *p* < 0.01 vs. the control group; * *p* < 0.05, ** *p* < 0.01 vs. the CCL2 group (mean ± S.D., *n* = 3).

As evidenced from immunofluorescence images, all the concentrations of *l*-CDL suppressed the activation of BV-microglia (*p* < 0.05) (Figure 7A,B). *l*-CDL (10 μM, 30 μM) groups and CCR2 antagonist RS504393 (10 μM) significantly inhibited the expression of CCR2 induced by CCL2 ( *p* < 0.05, *p* < 0.01) (Figure 7C). All concentration of l-CDL and RS504393 were reduced the level of TNF-α in cell culture supernatant (*p* < 0.05, *p* < 0.01) (Figure 7D). Besides, *l*-CDL at the concentration of 30 μM possessed stronger ability than RS504393 in reduction of IL-1β secretion (Figure 7E).

### 2.7. l-CDL Alleviated the Tumor Compression in the Sciatic Nerve

**Figure 8:**
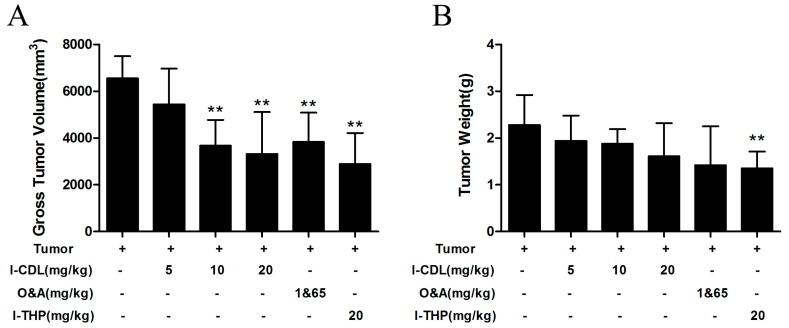
*l*-CDL alleviated the tumor compression in the sciatic nerve. (**A**) Effects of *l*-CDL on tumor volume; (**B**) Effects of *l*-CDL on tumor weight. ** *p* < 0.01 vs. the tumor group (mean ± S.D., *n* = 7).

To study the change of tumor cell after inoculation surgery, the size of tumor was measured by vernier caliper, weighed up the weight of tumor mass by electronic balance. According to the results, *l*-CDL at the dose of 10 mg/kg and 20 mg/kg showed stronger ability to decreased the volume of tumor (Figure 8A), and no significant change was observed in the weight of tumor mass in *l*-CDL treated groups compared with the tumor group (Figure 8B).

## 3. Discussion

The present study showed that *l*-CDL attenuated TCIP hypersensitivity, accompanying with downregulation of TNF-α and IL-1β expression levels and the decline of astrocytes and microglial activation, and it also decreased the expression of the mRNA and protein level for CCL2/CCR2. To elucidate the underlying mechanism of *l*-CDL on TCIP, we investigated the effect of *l*-CDL on glia in vitro. Our findings demonstrated that *l*-CDL could inhibit the activation of cytoactivity induced by TNF-α and the release of CCL2 in astrocytes, as well as the suppression of CCL2 induced BV-microglia activation through decreased its receptor-CCR2 level. Taken together, these data suggest that *l*-CDL alleviated TCIP in association with suppression of spinal proinflammatory cytokine and CCL2/CCR2 expression.

It is now well established that cancer pain, belongs to chronic pain [17], is developed due to tissue destruction, nerve compression, ischemia, and the release of cytokines and other inflammatory mediators [28] which result in spontaneous pain behaviour, heat hyperalgesia and mechanical allodynia over varying time courses. A report suggested that Meth-A sarcoma cells injected around mouse sciatic nerve induced a gradual development of spontaneous pain and thermal hyperalgesia by day 14. Mechanical sensitivity was gradually enhanced by day 10, but markedly decreased to below baseline by day 14 [4]. Interestingly, in the present study, a mouse model of TCIP was developed by inoculating S180 sarcoma cells to the immediate proximity of the sciatic nerve in BALB/c mice and observed a gradual intensification of mechanical sensitivity by day 14, which was similar to thermal hyperalgesia and spontaneous pain. The different reactivity on mechanical stimuli might be related with the types of sarcoma cells. All of these results demonstrated that gradual compression of sciatic nerve by malignant tumors resulted in pain hypersensitivity with this cancer pain mice model.

*l*-CDL, an alkaloid isolated from roots of *Corydalis chaerophylla* [29] and *l*-THP belong to an original berberine sinistral four hydrogen compounds. In the present study, we studied the efficacy of *l*-CDL against TCIP for the first time. Based on our study, treatment with *l*-CDL could attenuate pain hypersensitivity induced by tumor compression. It should be emphasized that *l*-CDL at the dose of 20 mg/kg possessed a stronger ability than O&A and *l*-THP in alleviating thermal hyperalgesia.

After the inoculation of cancer cells, inflammatory response was inevitable [3]. Activated microglia in the injured spinal cord produce various pro-inflammatory cytokines, proteases and other factors. Additionally, they recruited neural cells and immune cells from the periphery in order to respond to an injury of the spinal cord and central nervous system (CNS) [30]. In peripheral tissues, IL-1β and TNF-α are early mediators of innate immunity and inflammation. A recent study suggest that Lipoxins (LXs) and analogues exert strong analgesic effects on cancer-induced bone pain (CIBP) and suppress the pain associated expression of spinal proinflammatory cytokines (IL-1β and TNF-α) [31].

Our results showed that the levels of TNF-α and IL-1β protein and mRNA in serum and spinal cord were increased significantly in the tumor group compared with the sham group, which is consistent with the above study. Based on the expression of six inflammatory mediators following spinal cord injury (SCI) observed the second peak of IL-1 and TNF-α mRNA expression at day 14–28 [32]. *l*-CDL at the dose of 20 mg/kg showed a resemble power compared with O&A and *l*-THP in downregulating the TNF-α and IL-1β levels. In addition, a few studies suggest that TNF-α and IL-1β could stimulate astrocyte activation in the injured spinal cord [32,33]. Furthermore, in response to a nerve injury which occurred due to a tumor compression, the expression of cytokine may associate in astrocyte activation [34,35]. The present study further proved it. *l*-CDL could successfully suppress the TNF-α and IL-1β expression as mentioned which would subside the astrocyte activation.

Accumulating evidence suggests that activated glial cells contribute to the development of cancer pain [19,36]. Activated Schwann cells (SCs) in pancreatic cancer (PCa) recapitulate the hallmarks of ‘reactive gliosis’ and contribute to analgesia due to suppression of spinal glia [37]. The hydroalcoholic extract (50% HA) of Astragali radix relieves latinum-treated cancer pain and significantly decreases activation of microglia and astrocytes in the spinal cord and in brain areas [38]. IBA1 is probably the most widely used marker for microglial reaction in the pain field and it has been proved that GFAP^high^ reactive astrocytes regulate the chronic CNS inflammation [39]. In the present study, we observed that TCIP elicited microglia and astrocytes activation in the spinal dorsal horn on day 14. All doses of *l*-CDL lowered the expression of IBA1 and GFAP which was associated with inhibited spinal glia activation during TCIP, proving that the capability of *l*-CDL on reduction of TCIP.

Increasing evidence suggests that chemokines contribute to the pathogenesis of chronic pain via modulating glial activation and neural plasticity. CCL2 and CCR2 are the most well-studied chemokines for pain modulation [24]. Knock-out of CCR2 in both resident microglia and bone marrow-derived microglia (BMDM) in mice did not develop microglial activation or mechanical allodynia. Furthermore, treatment with MCP-1 neutralizing antibodies, as well as CCR2 antagonists, could prevent the microglial activation after nerve injury [40]. The CCR2 antagonist INCB3344 attenuated established mechanical allodynia in SN-CCI rats [41]. Our results showed that *l*-CDL could downregulate tumor compression-induced CCL2 and CCR2 protein and mRNA expression in spinal cord showing that *l*-CDL could decline mechanical allodynia as well as microglial activation. *l*-CDL at the dose of 20mg/kg showed stronger inhibitory ability than O&A and *l*-THP in TCIP model.

To identify the effect of *l*-CDL on CCL2/CCR2 dependent mechanisms that are involved in the regulation of glial activation, we carried out a in vitro experiment. TNF-α stimulated astrocytes [42] were employed to mimic in vivo neuroinflammatory microenvironment. RS504393, positive control of this study was a highly selective CCR2 chemokine receptor antagonist [23]. The major findings of the in vitro study include: (1) *l*-CDL could suppress TNF-α-induced astrocytes activation through downregulating the CCL2 and CCR2 expression and reduce IL-1β secretion as well; (2) *l*-CDL had no effect on IL-1β when CCR2 was silenced; (3) CCL2 induced BV-microglia activation accompanied with up-regulating the CCR2 and inflammatory factors level; (4) *l*-CDL suppressed the activation of BV-microglia activation after CCL2 stimulation through downregulating the CCR2 expression and inflammatory factors secretion. Besides, *l*-CDL (10 μM) presented comparable effect with RS504393. Based on the in vivo and in vitro study, we found that the attenuation of TCIP hypersensitivity by *l*-CDL could be due to CCL2/CCR2-mediated glial cells activation which was associated with anti-inflammatory effect.

The prevailing hypothesis put forward to explain cancer pain posits that cancers generate and secrete mediators which sensitize and activate primary afferent nociceptors in the cancer microenvironment [2]. Macrophages and microglia within the glioma microenvironment produce CCL2, which is critical for recruiting regulatory T cells (Treg) and myeloid-derived suppressor cells (MDSC) [43]. Additionally, tumor-derived angiogenesis induced by co-treatment of ganglioside GM1 and macrophages was reduced by the addition of the CCR2 antagonist RS102895 [44]. These evidences further verify that CCL2/CCR2 signaling is involved in the tumor microenvironment, which explained why *l*-CDL decreased the volume of tumor and diminished angiogenesis, and alleviated the TCIP after sciatic nerve injury. It is postulated that *l*-CDL alleviated TCIP via CCL2/CCR2 pathway.

In conclusion, the current study exhibited that *l*-CDL attenuated TCIP hypersensitivity, and it showed a strong ability to modulate inflammatory response. Additionally, the present study revealed that CCL2/CCR2-mediated astroglial and microglia activation may be the vital mechanism of *l*-CDL on TCIP. Therefore, all the facts confirming that, *l*-CDL is a prospective agent in the management of TCIP.

## 4. Materials and Methods

### 4.1. Animals

Male BALB/c mice (25–30 g) were provided by Comparative Medical Center of Yangzhou University and caged in groups of seven prior to any procedure, and individually after procedures. Mice were maintained on a 12:12 h dark-light cycle with food and water ad libitum. All protocols were conducted according to the Guidelines of the National Institutes of Health and the International Association for the Study of Pain. Meanwhile, all experiments were carried out in accordance with Guidelines for the Care and Use of Laboratory Animals and ethical approval was provided by the Animal Care and Use Committee of China Pharmaceutical University.

### 4.2. Tumor Inoculation

Murine sarcoma cell line S180 were obtained from the Shanghai Institute of Biochemistry and Cell Biology, Chinese Academy of Sciences, Cell Bank. Cells were incubated with Roswell Park Memorial Institute (RPMI) 1640 medium and supplemented with 10% newborn calf serum in a humidified 95% air and 5% CO_2_ at 37 °C. S180 sarcoma cells were carried intraperitoneally in carrier mice. The ascites of the carrier mouse was aspirated under sodium pentobarbital and the number of tumor cells was counted by a hemacytometer. Ascites tumor cells resuspended in PBS in a concentration of 2 × 10^6^ cells/mL. Tumor cells were injected to the muscular tissue proximity of the nerve near the trochanter, immediately distal to where the posterior biceps semitendinosus branches off the common sciatic nerve. The wound was closed in layers. All these procedures were followed as mentioned in Shimoyama with few modifications [4]. Apart from the sham group, inoculation surgery was carried out for all other groups to perform the neuropathic pain on sciatic nerve. The surgery was conducted as described previously. For the sham group, the same volume of saline was injected instead of ascites.

### 4.3. Drugs and Administration

*l*-CDL and *l*-THP were prepared by the School of Traditional Chinese Medicine, China Pharmaceutical University. O&A was purchased from China National Pharmaceutical Industry Corporation Ltd. (Beijing, China). CCL2 and RS504393 (CCR2 inhibitor) were purchased from R&D System, Inc. (Suite 302, Minneapolis, MN, USA). *l*-THP and O&A were dissolved in sodium carboxyl methyl cellulose (CMC-Na). The group protocols are shown in Table 1. All drug doses were based on the results of preliminary experiments. Treatment protocol: After tumor inoculation surgery was performed and all groups had a tumor mass detectable by tactile sensation with the fingers (day 6), CMC-Na or drugs was administered intragastrically for 9 days (from day 6 to day 14) once per day. On day 14, all animals were sacrificed after behavioral experiments.

### 4.4. Gross Behavior

To assess spontaneous pain, mice were observed on the time course of cumulative lifting duration of the ipsilateral paw after inoculation. After intragastric administration of drugs or CMC-Na, mice were placed in a clear plastic chamber with a glass floor to acclimate for a period of approximately 20 min. The cumulative duration of hind paw-lifting during a 10 min observation period was measured. The lifting of the paw as a part of grooming behavior was not taken into account. The assessments were made prior to and on day 6, 10 and 14 after surgery [4].

### 4.5. Assessment of Thermal Hyperalgesia

Paw withdrawal latency to radiant heat stimulation was measured in each paw before the surgery and on day 6, 10 and 14 after inoculation. When radiant heat was applied, mice were examined to make sure that the area of the paw was in contact with the glass floor. Paw withdrawal latency was determined as a mean of two measurements per paw. The intensity of the radiant heat was adjusted to ensure the paw withdrawal latency of sham group falls in the range of 10 ± 2 s through [45].

### 4.6. Assessment of Mechanical Allodynia

Mechanical allodynia was evaluated on the day before the surgery and on day 6, 10 and 14 after inoculation. Mice were placed in suspended cages with a wire mesh bottom about 30 min for acclimation and a set of eight calibrated Von Frey fibers (ranging from 0.016 to 1.40 g of force) were applied to the plantar surface of the hindpaw until they bent. Mechanical allodynia was measured with calibrated Von Frey fibers using the up-down method [46].

### 4.7. Cell Cultures

Primary mouse astrocytes were prepared as previously described [47] with minimal modification. Briefly, the spinal cord was isolated from neonatal (P1–P2) mice. The meninges were removed, and spinal cord strips were rinsed three times with cold D-Hank’s buffered saline. Then, the strips were minced with sterile scissors and digested with 0.25% Trypsin solution for 10 min at 37 °C. Trypsinization was stopped by adding an equal volume of culture medium, which was Dulbecco’s modified Eagle’s medium (DMEM) containing 10% fetal calf serum (FCS). Cells were cultured in a humidified 95% air and 5% CO_2_ at 37 °C. The medium was replaced every three days with 10% FCS. Cells were allowed to grow about two weeks until 90 % confluent, then shook for 5 h at 150 rpm to detach cells sitting on top of the astrocyte monolayer and then re-plated to culture flask for western blotting analysis, 6-well plates for ELISA assay, and lab dishes for immunofluorescence assay. Astrocytes were incubated with TNF-α for two hours, then treated with *l*-CDL (3 μM, 10 μM, 30 μM) for two hours. BV-microglia were obtained from the Cell Bank of the Shanghai Institute of Biochemistry and Cell Biology, Chinese Academy of Sciences. To determine the expression of CCR2, the treatment of CCL2 was done two hours prior to *l*-CDL (3 μM, 10 μM, 30 μM) treatment. After the treatment, the cells were collected for immunofluorescence or Western blotting assay. Culture supernatant were collected for ELISA assay.

### 4.8. Immunofluorescence

On Day 14 after inoculation, animals were deeply anesthetized with sodium pentobarbital and perfused through the ascending aorta with PBS, followed by 4% paraformaldehyde in 0.01 M PBS. After the perfusion, the spinal cord segments (L4–L6) were removed and post-fixed overnight in the fixative solution, then cryoprotected overnight in a solution of 30% sucrose in PBS. Frozen spinal cord tissues, which were embedded in TissueTek OCT compound, were cut into 25 μm sections on a cryostat. Then, these sections were collected in PBS for immunofluorescence as previously described [45]. Sections were incubated in a blocking solution that consisted of 10% normal goat serum and 0.3% Triton-X100 in PBS for 30 min at room temperature. The sections were then incubated overnight at 4 °C with IBA-1 antibody (1:100, rabbit; Beijing Biosynthesis Biotechnology Co., Ltd., Beijing, China) and GFAP antibody (1:100, rabbit; Beijing Biosynthesis Biotechnology Co., Ltd.). Then, the sections were incubated with Goat Anti-rabbit IgG (Beijing Biosynthesis Biotechnology Co., Ltd., Beijing, China) for 2 h at room temperature, and the nuclei were stained with DAPI. The stained sections were examined with a laser scanning confocal microscope (Carl Zeiss, Oberkochen, Germany) and images were taken with a CCD Spot camera equipped with image acquisition software (Axio Vision; Carl Zeiss Oberkochen, Germany). To assess glia activation, cultured astrocytes and BV-microglial cells were fixed with 4% paraformaldehyde solution for 30 min and then processed as mentioned above.

### 4.9. RT-PCR

The total RNA of L4–L6 spinal cord segments were extracted using Trizol reagent (Takara Bio Inc., Shiga, Japan). RNA quantity and purity were determined using an ultraviolet spectrophotometer (Beijing Purkinje General Instrument Co., Ltd. Beijing, China). RT-PCR analysis was performed in the Real-time Detection System (Rotor-Gene 6000, Hamburg, Germany) by SYBR Premix Ex Taq™ II kit (Takara Bio Inc.). The cDNA was amplified with the following primers: CCL2 forward, 5′-AGC AGC AGG TGT CCC AAA GA-3′; CCL2 reverse, 5′-GTG CTG AAG ACC TTA GGG CAG A-3′; CCR2 forward, 5′-AAG TTC AGC TGC CTG CAA AGA C-3′; CCR2 reverse, 5′-TCA TCA AGC TCT TGG ATA CTT CGT G-3′; TNF-α forward, 5′-CCT CAC ACT CAC AAA CCA CCA-3′, TNF-α reverse, 5′-ACA AGG TAC AAC CCA TCG GC-3′; IL-1β forward, 5′-ATG CCA CCT TTT GAC AGT GAT G-3′, IL-1β reverse, 5′-TGA TGT GCT GCT GCG AGA TT-3′, Glyceraldehyde 3-phosphate dehydrogenase (GAPDH) forward, 5′-TGT TCC TAC CCC CAA TGT G-3′; GAPDH reverse, 5′-GTG TAG CCC AAG ATG CCC T-3′. The PCR amplifications were performed at 95 °C for 30 s, followed by 40 cycles of thermal cycling at 95 °C for 5 s, and 60 °C for 45 s. Data were collected after each cycle and displayed graphically. Melt curves were performed on completion of the cycles to ensure that nonspecific products were absent. Quantification was performed by normalizing cycle threshold (*C*_t_) values with GAPDH *C*_t_ and analyzed with the 2^–ΔΔ*C*t^ method.

### 4.10. Western Blot

Spinal cord tissues were collected from deeply anesthetized animals perfusing transcardially with 50 mL of 0.9% NaCl on day 14. Protein concentrations were determined by BCA Protein Assay (Applygen, Beijing, China). Proteins (50 μg) were loaded for each lane and separated with sodium dodecyl sulfate polyacrylamide gel electrophoresis. The proteins were then transferred and incubated overnight at 4 °C with Anti-MCP1 (CCL2) antibody (ab25124) (1:1000, mouse; Abcam, Cambrige, MA, USA.), CCR2 antibody (36784) (mouse, 1:1000, mouse; Signalway Antibody LLC, College Park, MD, USA.); GAPDH antibody (1:10,000, mouse; Sigma-Aldrich Co. LLC., (St. Louis, MO, USA), was used as loading control. These blots were further incubated with horseradish peroxidase–conjugated secondary antibody, developed in enhanced chemiluminescence solution, and exposed on Hyperfilm (Bio-Rad, Hercules, CA, USA,) for 1 to 5 min. Specific bands were evaluated by apparent molecular size. The intensity of the selected bands was analyzed using ImageJ software (National Institutes of Health, Bethesda, MD, USA). Cellular proteins were extracted from the primary microglial cells and astrocytes using RIPA buffer. The homogenates were centrifuged for 15 min at 12,000× *g* at 4 °C. The quantity of protein in each supernatant was determined using a BCA protein assay kit and then processed as shown above.

### 4.11. ELISA

CCR2, TNF-α and IL-1β ELISA kits were purchased from Cloud-Clone Corp. (Katy, TX, USA). Blood samples for ELISA analysis were collected form animals before the sacrifice. After animals were sacrificed, L4–L6 spinal cord segments were collected and homogenized for ELISA analysis. Treated glial cells and culture supernatant were collected and homogenized and measured the expression of CCR2, TNF-α and IL-1β by ELISA kits. ELISA was performed in accordance with the instructions of the manufacturer.

### 4.12. Statistical Analysis

All experiments were repeated at least three times, and performed in a parallel manner unless otherwise indicated. The behavioral was analyzed by two-way ANOVA followed by the Tukey’s post hoc test. All data were expressed as mean ± SD (standard deviation). Differences between two groups were compared using Student’s *t*-test and significance of difference is indicated as *p* < 0.05.

## 5. Conclusions

*l*-CDL can effectively attenuate TCIP hypersensitivity, and modulate inflammatory response significantly. Further, the present study revealed that the vital mechanism of *l*-CDL may be via the CCL2/CCR2-mediated astroglial and microglia activation on TCIP. Therefore, all the facts confirming that, *l*-CDL is a potential agent in the management of TCIP.

## Figures and Tables

**Table 1:** The experimental groups.

Group	Drug Dose	*n*	Surgery
Sham	-	7	-
Tumor	-	7	Tumor inoculation
*l*-CDL	5 mg/kg	7	Tumor inoculation
	10 mg/kg	7	Tumor inoculation
	20 mg/kg	7	Tumor inoculation
O&A	1&65 mg/kg	7	Tumor inoculation
*l*-THP	20 mg/kg	7	Tumor inoculation
